# The COVID-19 Vaccination Coverage in ICU Patients with Severe COVID-19 Infection in a Country with Low Vaccination Coverage—A National Retrospective Analysis

**DOI:** 10.3390/jcm12051749

**Published:** 2023-02-22

**Authors:** Liana Valeanu, Stefan Andrei, Bianca Morosanu, Dan Longrois, Serban-Ion Bubenek-Turconi, COVATI-RO Collaborative

**Affiliations:** 1 Cardiac Anesthesiology and Intensive Care Department I, Emergency Institute for Cardiovascular Diseases C. C. Iliescu, 258 Fundeni Road, 022328 Bucharest, Romania; 2 Anesthesiology and Intensive Care Department, Carol Davila University of Medicine and Pharmacy, 8 Eroii Sanitari Blvd., 050474 Bucharest, Romania; 3 Department of Anesthesiology and Intensive Care, Bichat-Claude Bernard University Hospital, Sorbonne Universités, INSERM UMR 1148, 46 Rue Henri Huchard, 75018 Paris, France

**Keywords:** vaccination, ICU, COVID-19, survival

## Abstract

Background: Romania is one of the European countries with low COVID-19 vaccination coverage. The main goal of this study was to describe the COVID-19 vaccination status in patients admitted to Romanian ICUs with a severe COVID-19 infection. The study describes the patients’ characteristics according to their vaccination status and evaluates the association between vaccination status and ICU mortality. Methods: This retrospective, observational, multicenter study included patients with confirmed vaccination status admitted to Romanian ICUs from January 2021 to March 2022. Results: Two thousand, two hundred and twenty-two patients with confirmed vaccination status were included. Five point one three percent of patients were vaccinated with two vaccine doses and one point seventeen percent of patients were vaccinated with one vaccine dose. The vaccinated patients showed a higher rate of comorbidities but had similar clinical characteristics at ICU admission and lower mortality rates compared to non-vaccinated patients. Vaccinated status and higher Glasgow Coma Scale at ICU admission were independently associated with ICU survival. Ischemic heart disease, chronic kidney disease, higher SOFA score at ICU admission and the need for mechanical ventilation in ICU were independently associated with ICU mortality. Conclusion: Lower rates of ICU admission were observed in fully vaccinated patients even in a country with low vaccination coverage. The ICU mortality was lower for fully vaccinated patients compared to non-vaccinated patients. The benefit of vaccination on ICU survival could be more important in patients with associated comorbidities.

## 1. Introduction

The COVID-19 pandemic has put extreme pressure on medical systems worldwide. Initial social distancing, quarantine, and lockdown measures were effective in limiting viral transmission, but were impossible to maintain indefinitely as social and economic crises were developing [1]. In the long run, global immunity achieved by vaccination rather than herd immunity seemed to be the viable solution.

COVID-19 vaccines were available less than 10 months after the start of the pandemics. By the end of 2021, two mRNA COVID-19 vaccines (BNT162b2, BioNTech/Pfizer, Germany, Mainz/New York, NY, USA and mRNA-1273, Moderna, Cambridge, MA, USA) and two adenoviral vector COVID-19 vaccines (AZD1222, Oxford/AstraZeneca, UK/Sweden and Ad26.COV2.S, Janssen/Johnson&Johnson, Leiden, Netherlands/New Brunswich, USA) were approved in the European Union. Almost one billion doses of vaccine were used in the European Economic Area in 2021 [2]. Clinical trials and reports from various countries showed that COVID-19 vaccines are effective against viral transmission, symptomatic, and severe COVID-19 infection [3,4,5,6]. Moreover, the risk of severe disease, hospitalization, and death remains significantly reduced for novel SARS-CoV-2 variants [7,8,9,10].

Vaccination rates vary tremendously among different European countries, despite similar availability of vaccine doses. Western European countries reached vaccination rates of between 60 and 80% or even higher than 80% for the Iberian countries and Malta by the end of 2021. While Central European countries had vaccination rates of between 40 and 60%, Eastern European countries such as Romania and Bulgaria did not manage to achieve more than 40% vaccination coverage [7,11].

During the surges of the COVID-19 pandemic, the Romanian ICUs faced extreme pressure due to a shortage of equipment and medical personal. The overall mortality of ICU patients admitted with SARS-CoV-2 infection before vaccines were available was 62% [12]. Patients older than 80 years had higher mortality rates, especially if intubated [13]. The almost two-fold increase in total cost for ICU hospitalization during pandemic waves nearly caused the chronically underfinanced medical system to collapse [14], while elective surgical activity decreased [15].

The study’s main objective was to comparatively describe the characteristics of patients who were admitted to the ICU for severe SARS-CoV-2 infection, according to their vaccination status. The secondary objectives were (i) to describe the COVID-19 vaccination status in patients admitted to Romanian ICUs with severe COVID-19 infection and (ii) to evaluate the association between vaccination status and ICU mortality.

## 2. Materials and Methods

### 2.1. Patients, Data Collection, and Study Design

This retrospective, observational, multicenter study was conducted in ICUs at a national level. Data were collected anonymously by the treating intensivists using an online platform at the initiative of the Romanian Society of Anaesthesia and Intensive Care. A total of 42 ICUs from university, county, and municipal hospitals across the country participated in this study. Patients with severe SARS-CoV-2 infection admitted to Romanian ICUs from January 2021 to March 2022 were included. For each patient, data about age, gender, associated medical history, vaccination status, clinical severity at the moment of ICU admission, and the need for invasive mechanical ventilation (MV) were retrospectively collected. The patients’ outcomes were dichotomized as survivors (discharged from ICU) or as deceased (ICU death). For study analyses, we considered the patients who had received two vaccine doses to be fully vaccinated. Patients who received one vaccine dose were excluded from the statistical analysis because it was unclear if they were fully vaccinated with a one-dose COVID-19 vaccine or incompletely vaccinated with only one dose of a two-dose COVID-19 vaccine.

The circulating variants of SARS-CoV-2 were not determined for each patient. However, we arbitrarily classified the patients into four periods of surge (A, B, C, D) according to the hospitalization month, based on the official data of the Romanian Government [16]: period A (from January to February 2021, first cases of Alpha variant); period B (from March to June 2021, predominantly Alpha variant); period C (from July to December 2021, predominantly Delta variant); and period D (from January to March 2022, end of data collection) predominantly Omicron variant).

### 2.2. Ethics

The ethical approval for this study was granted by the Department of Medical Education and Research of the Romanian Society of Anaesthesia and Intensive Care (decision number 7, 4 April 2022). Considering its retrospective, observational design and completely anonymized data collection, the patient’s consent for data inclusion and use was waived by the ethics committee. 

### 2.3. Statistical Analysis

The patients with complete datasets were included in statistical analysis. Numerical variables were presented as mean ± standard deviation or as median with [25–75%] interquartile range (IQR), as appropriate. The parametric distribution was checked using histograms and using the Shapiro–Wilk’s test. The continuous variables were compared using the Student’s test or Mann–Whitney U test, as appropriate. Categorical variables were presented as numbers (percentages). The comparisons between categorical variables were performed using the Chi-square test. 

The main study endpoint (the vaccination rate) was descriptively presented as a percentage (95% CI). The secondary endpoints were statistically managed as follows:

(1) The characteristics of vaccinated and non-vaccinated patients were comparatively presented using appropriate statistical tests (Student, Mann–Whitney U, or Chi-square);
(2) The association between vaccination status and ICU mortality was evaluated using a logistic regression approach. Non-redundant variables with clinical pertinence or statistical significance in univariate logistic regression analyses (*p* < 0.05) were introduced in the multivariate logistic model. The validity conditions for logistic regression were verified in order to have at least 10 events for each variable included in the multivariate logistic model.

A bilateral *p* < 0.05 was, a priori, considered statistically significant. The statistical analyses were performed using RStudio (Version 1.1.447—© 2009–2018 RStudio, Inc., Wien, Austria).

## 3. Results

### 3.1. Vaccination Rate in Romanian ICUs

**Figure 1:**
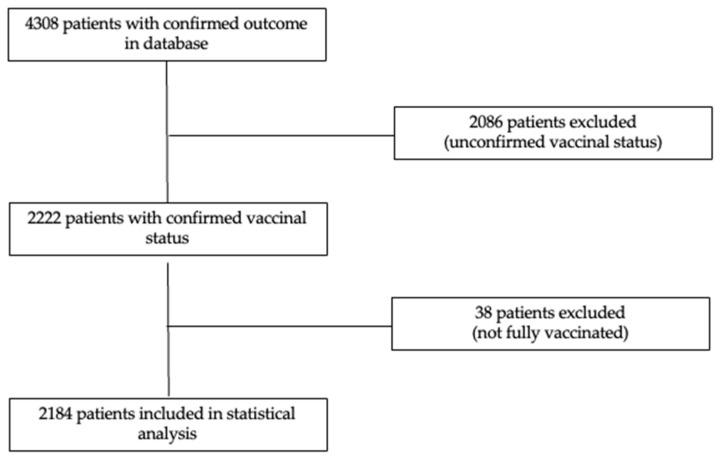
Study flowchart.

Two thousand, two hundred and twenty-two patients with confirmed vaccination status were included. From this cohort, only 114 patients were vaccinated with two doses, corresponding to a vaccination rate of 5.13%, 95% CI (4.25%; 6.13%). Thirty-eight (1.71%) patients received only one vaccine dose and were further excluded from the statistical analysis (Figure 1).

### 3.2. Characteristics of Fully Vaccinated Patients Admitted in ICU

The collected patients’ characteristics according to their vaccination status are presented in Table 1.

The vaccinated patients showed a higher rate of ischemic heart disease (36% vs. 25%, *p* = 0.012), chronic kidney disease (21% vs. 12%, *p* = 0.003), and chronic dialysis (5% vs. 1%, *p* < 0.001) as associated comorbidities, compared to the non-vaccinated patients. The age, gender, and rate of the other collected comorbidities were not statistically different between the groups of patients. However, the vaccinated patients had a higher median number of comorbidities (2 [1,2]) compared to the non-vaccinated patients (2 [1–3], *p* = 0.016). The number of patients with more than four cumulated comorbidities was higher in the vaccinated group. The clinical status and severity were similar between the two groups regarding the initial ICU admission (SOFA score, dyspnea, fever, ARDS, need for MV) and management, excepting the neurological status (Table 1). The non-vaccinated patients had a lower Glasgow Coma Scale (GCS) compared to the vaccinated group (15 [7–15] vs. 15 [12–15], *p* = 0.010).

Mortality was lower for vaccinated patients compared to non-vaccinated patients (58% vs. 67%, *p* = 0.045), while the ICU length of stay was similar between vaccinated and non-vaccinated patients.

### 3.3. Risk Factors for ICU Mortality

The results of the univariate logistic regression analysis with ICU mortality as the dependent variable are presented in Table 2. Several variables were associated with ICU mortality in univariate logistic regression analyses: high blood pressure, OR 2, 95% CI (1.7–2.4), *p* < 0.001; ischemic heart disease, OR 2.1, 95% CI (1.7–2.6), *p* < 0.001; chronic heart failure, OR 1.8, 95% CI (1.4–2.3), *p* < 0.001; diabetes, OR 1.7, 95% CI (1.4–2), *p* < 0.001, chronic kidney disease, OR 2.7, 95% CI (1.9–3.7), *p* < 0.001; chronic obstructive pulmonary disease, OR 1.6, 95% CI (1.05–2.5), *p* = 0.030; past or current neoplasia, OR 2.1, 95% CI (1.4–3.1), *p* = 0.001; the SOFA score on ICU admission, OR 1.08, 95% CI (1.06–1.1), *p* < 0.001; the need for non-invasive ventilation, OR 2.5, 95% CI (2–2.9), *p* < 0.001; and the need for invasive mechanical ventilation, OR 22, 95% CI (17–28), *p* < 0.001. The vaccinated status, OR 0.68, 95% CI (0.46–0.99), *p* = 0.046, higher GCS on ICU admission, OR 0.8, 95% CI (0.77–0.82), *p* < 0.001, and COVID-19 period D, OR 0.03, 95% CI (0.01–0.25), *p* = 0.001 were inversely associated with ICU mortality.

The multivariate logistic regression analysis with ICU mortality as the dependent variable (Table 2) found that the vaccinated status, OR 0.54, 95% CI (0.31–0.93), *p* = 0.027, and higher GCS on admission, OR 0.9, 95% CI (0.85–0.92), *p* < 0.001 were factors independently associated with ICU survival. The same multivariate logistic regression analysis showed that ischemic heart disease, OR 1.9, 95% CI (1.4–2.6), *p* < 0.001, chronic kidney disease, OR 2.2, 95% CI (1.5–3.3), *p* < 0.001, admission SOFA, OR 1.03, 95% CI (1.01–1.06), *p* = 0.003, the need for non-invasive ventilation, OR 2, 95% CI (1.6–2.7), *p* < 0.001, and the need for invasive mechanical ventilation, OR 14, 95% CI (11–18), *p* < 0.001 were factors independently associated with ICU mortality.

## 4. Discussion

Our study found a very low rate of vaccination in ICU patients admitted for severe COVID-19 infection. Only 5.13% of patients were vaccinated with two doses and 1.71% of patients received one dose of the COVID-19 vaccine. This low vaccination rate in ICU patients can be partially explained by the low national vaccination coverage. COVID-19 vaccines were available in Romania at the end of December 2020. The national vaccination strategy focused on healthcare providers and individuals at high risk of complications following COVID-19 infection during the first weeks. After 15th March 2021, the vaccination was available to the general population. By the end of March 2021 (period B), only 5.43% of the Romanian population was fully vaccinated, but the vaccination rate increased slowly to 23.69% at the end of June 2021 (period B), 40.91% at the end of December 2021 (period C), and 42.26% in March 2022 (period D) [18]. This low national vaccination rate is mainly explained by the hesitation of the population to vaccinate, caused by conspiracy theories, lack of medical education, fear of adverse effects, and the belief that vaccines are not effective [19,20]. Although the COVID-19 vaccination campaign received extensive media coverage and was publicly promoted by medical professionals as well as opinion leaders, it did not impact vaccination rates as expected. Even though the vaccination rate was globally low in the Romanian population during the study period, the rate of vaccination in ICU patients was strikingly low. Previous studies already report that COVID-19 vaccination reduces the risk of severe symptoms, hospitalization [21], ARDS, and the need for invasive MV [22,23]. Our findings are coherent with other reports that advocated a lower rate of ICU admission in vaccinated patients [23,24,25,26,27,28,29], although in this cohort there was no difference in the incidence of ARDS or the need for invasive MV between unvaccinated and fully vaccinated patients.

The vaccinated patients with severe COVID-19 infection admitted to ICU presented a higher rate of ischemic heart disease, chronic kidney disease, chronic dialysis, and a higher cumulated number of associated medical conditions. These results are similar to other studies that report a notably higher incidence of comorbidities in vaccinated patients requiring ICU admission [22,30,31,32,33,34,35] and a weaker association between vaccination and reduced risk of hospitalization for patients with impaired immune status [22]. The high rate of comorbidities might be explained by the exacerbation of coexisting diseases requiring ICU hospitalization or by lower vaccine effectiveness in this population [32,33]. 

The mortality rate was lower for vaccinated patients compared to non-vaccinated patients in this cohort. The vaccinated status was statistically associated with survival for the patients in this cohort, after adjustment on age, gender, comorbidities, and clinical severity. This finding is in line with other studies from countries with high or low vaccination coverage that report lower mortality rates in vaccinated patients [24,25,26,27,29,30,31,32,36,37,38]. Moreover, full vaccination status is associated with lower mortality among intubated patients with COVID-19-related ARDS, even in the presence of comorbidities [34], suggesting an immunity advantage in the case of severe infection [39] in the population with the highest case fatality rates [40,41]. Another study reports no difference in ICU mortality between vaccinated and non-vaccinated patients [33], but in that cohort vaccinated patients had significantly higher rates of immunosuppression or comorbidities compared to non-vaccinated patients. Although no causality could be affirmed, considering the lower mortality observed in vaccinated patients despite the higher incidence of comorbidities, the population at risk might supplementarily benefit from further prevention measures or interventions such as vaccine boosters [33,34]. The perspective of reinfection as new variants of COVID-19 emerges and the higher rate of ICU admission and mortality in cases of reinfection [42], combined with the waning effects of vaccine protection with the increasing time interval since vaccination [43], supplementary support immunization practices. Although there was no statistically significant difference in the ICU length of stay between vaccinated and non-vaccinated patients, the shorter ICU length of stay in the latter group might have been due to a higher mortality rate in non-vaccinated patients.

### 4.1. Relevance of Our Results

The findings of this study underline once more the importance of vaccination, particularly in patients with a higher number of comorbidities and in countries with underserved ICU and healthcare systems. To limit mortality, national strategies addressing the population’s hesitancy to vaccinate should be developed while preparing the healthcare system to successfully respond in case of following COVID-19 waves.

### 4.2. Limitations

This study can be discussed on several points. Considering the retrospective, non-randomized study protocol, the results should be taken with caution and no causality can be firmly stated. We did not collect all the variables potentially associated with mortality (detailed comorbidities, presence of immunosuppression, length of hospital stay before ICU admission, ICU management and complications, need for vasopressor therapy, cause of death, centre-effect, etc.) and we did not describe or analyze the time lapse between full vaccination and COVID-19 infection. 

Furthermore, the type of vaccination for each patient was not collected and patients fully vaccinated with a one-dose vaccine were excluded from the analysis. Further studies are necessary to confirm the positive effect of vaccination on ICU survival, especially in patients with coexisting diseases.

## 5. Conclusions

The COVID-19 vaccination rate in ICU patients was very low, even in a population with low vaccination coverage. Patients with coexisting diseases may supplementarily benefit from the protective effect of vaccination against COVID-19 regarding ICU admission and mortality.

## Figures and Tables

**Table 1:** Patients’ characteristics. Non-vaccinated versus fully vaccinated.

Variables	Non-Vaccinated (*n* = 2070)	Fully Vaccinated (*n* = 114)	*p*-Value
Age (years), median [IQR]	68 [59–75]	66 [56–72]	0.057
Male gender, *n* (%)	1077 (52)	51 (45)	0.129
ICU admission and management			
Fever (yes), *n* (%)	736 (36)	34 (30)	0.212
Dyspnea (yes), *n* (%)	1489 (72)	84 (84)	0.685
ARDS (yes), *n* (%)	938 (41)	43 (38)	0.558
Invasive mechanical ventilation (yes), *n* (%)	1157 (56)	60 (53)	0.495
Non-invasive ventilation (yes), *n* (%)	1277 (62)	64 (56)	0.236
High flow nasal cannula oxygen therapy (yes), *n* (%)	993 (48)	50 (44)	0.392
GCS, median [IQR]	15 [7–15]	15 [12–15]	0.010
SOFA, median [IQR]	7 [4–12]	8 [4–12]	0.202
Corticosteroid usage, *n* (%)	1788 (86)	87 (76)	0.003
Received remdesivir (yes), *n* (%)	828 (40)	42 (37)	0.503
Associated medical conditions			
Ischemic heart disease (yes), *n* (%)	526 (25)	41 (36)	0.012
Autoimmune disease (yes), *n* (%)	49 (2)	6 (5)	0.055
Dialysis patient (yes), *n* (%)	16 (1)	6 (5)	<0.001
COPD (yes), *n* (%)	111 (5)	5 (4)	0.651
Past or current cancer (yes), *n* (%)	136 (7)	12 (11)	0.102
Chronic kidney disease ^1^ (yes), *n* (%)	244 (12)	24 (21)	0.003
Diabetes (yes), *n* (%)	640 (31)	44 (39)	0.085
Heart failure (yes), *n* (%)	354 (17)	21 (18)	0.716
Arterial hypertension (yes), *n* (%)	1328 (64)	78 (68)	0.354
Number of comorbidities, median [IQR]	2 [1–2]	2 [1–3]	0.016
Comorbidities, *n* (%)			<0.001
- No comorbidity (yes), *n* (%)	455 (22)	25 (22)	
- 1 comorbidity (yes), *n* (%)	564 (27)	21 (18)	
- 2 comorbidities (yes), *n* (%)	563 (27)	27 (24)	
- 3 comorbidities (yes), *n* (%)	295 (14)	19 (17)	
- 4 comorbidities (yes), *n* (%)	145 (7)	9 (8)	
- 5 comorbidities (yes), *n* (%)	39 (2)	9 (8)	
- 6 comorbidities (yes), *n* (%)	9 (0.4)	4 (4)	
COVID-19 period, *n* (%)			<0.001
- A (01–02/21)	136 (7)	4 (4)	0.240
- B (03–06/21)	1169 (57)	17 (15)	<0.001
- C (07–12/21)	752 (36)	90 (79)	<0.001
- D (01–03/22)	13 (0.6)	3 (3)	0.047
Outcomes			
ICU Death, *n* (%)	1387 (67)	66 (58)	0.045
- No comorbidities	206 (10)	9 (8)	-
- With comorbidities	1181 (57)	57 (50)	-
ICU LOS (days), median [IQR]	7 [4–12]	8 [5–16]	0.057

^1^ Chronic kidney disease was defined as creatinine clearance <60 mL/min [17]. Abbreviations: IQR—interquartile range; ICU-Intensive care unit; ARDS—acute respiratory distress syndrome; GCS—Glasgow coma scale; SOFA—sequential organ failure assessment score; COPD—chronic obstructive pulmonary disease; LOS—length of stay.

**Table 2:** Univariate and multivariate logistic regression analysis with ICU mortality as the dependent variable.

Variables	Univariate Analysis		Multivariate Analysis	
	OR (95% CI)	*p*-Value	OR (95% CI)	*p*-Value
Study endpoint				
Vaccinated (yes)	0.68 (0.46–0.99)	0.046	0.54 (0.31–0.93)	0.027
Patients’ baseline characteristics				
Age (year)	1 (0.99–1)	0.777		
Gender (male)	1.18 (0.98–1.4)	0.076		
COVID-19 period				
- A (01–02/21)	comparator			
- B (03–06/21)	1.02 (0.7–1.5)	0.900	1.2 (0.7–2)	0.485
- C (07–12/21)	0.90 (0.6–1.3)	0.489	1.3 (0.8–2.3)	0.262
- D (01–03/22)	0.03 (0.01–0.25)	0.001	0.1 (0.01–1.6)	0.107
Comorbidities				
AHT (yes)	2 (1.7–2.4)	<0.001	1.2 (0.91–1.6)	0.205
IHD (yes)	2.1 (1.7–2.6)	<0.001	1.9 (1.4–2.6)	<0.001
CHF (yes)	1.8 (1.4–2.3)	<0.001	1 (0.7–1.5)	0.857
Diabetes (yes)	1.7 (1.4–2)	<0.001	1.3 (0.99–1.7)	0.056
Autoimmunity (yes)	1.13 (0.63–2)	0.684		
CKD (yes)	2.7 (1.9–3.7)	<0.001	2.2 (1.5–3.3)	<0.001
Chronic dialysis (yes)	2.3 (0.8–6.7)	0.137		
COPD (yes)	1.6 (1.05–2.5)	0.030	1.6 (0.91–2.7)	0.104
Past or current neoplasia (yes)	2.1 (1.4–3.1)	0.001	1.6 (0.92–2.7)	0.101
ICU admission and evolution				
Fever (yes)	1.1 (0.9–1.3)	0.356		
Dyspnea (yes)	0.96 (0.8–1.2)	0.955		
SOFA score (each score point)	1.08 (1.06–1.1)	<0.001	1.03 (1.01–1.06)	0.003
GCS (each score point)	0.8 (0.77–0.82)	<0.001	0.9 (0.85–0.92)	<0.001
Need for non-invasive ventilation (yes)	2.5 (2–2.9)	<0.001	2 (1.6–2.7)	<0.001
Need for invasive mechanical ventilation (yes)	22 (17–28)	<0.001	14 (11–18)	<0.001
Corticosteroid usage	2.2 (1.8–2.8)	<0.001	0.9 (0.6–1.2)	0.475
Received remdesivir	1.2 (0.98–1.4)	0.076		

Abbreviations: AHT—arterial hypertension, IHD—ischemic heart disease, CHF—chronic heart failure, CKD—chronic kidney disease, COPD—chronic obstructive pulmonary disease, SOFA—sequential organ failure assessment, GCS—Glasgow coma scale, ICU—intensive care unit.

## Data Availability

Restrictions apply to the availability of these data. The data presented in this study are available on reasonable request from the corresponding author.

## Supplementary Materials

The following supporting information can be downloaded at: https://www.mdpi.com/article/10.3390/jcm12051749/s1, COVATI-RO Collaborative: collaborators, affiliations.
