# Supplementary material for: The COVID-19 Vaccination Coverage in ICU Patients with Severe COVID-19 Infection in a Country with Low Vaccination Coverage—A National Retrospective Analysis
Source: J Clin Med. 2023 Feb 22;12(5):1749. doi: 10.3390/jcm12051749 (PMC10003614; doi:10.3390/jcm12051749)
Supplement: Supplementary file 1 [file jcm-12-01749-s001.zip › jcm-2188254-supplementary.pdf]

COVATI-RO Collaborative
Collaborators, Affiliations

Ioana-Marina Grințescu[1,2], Liliana Mirea[1,2], Raluca Ungureanu[1,2], Cristian Cobilinschi[1,2], Ana-Maria Cotae[1,2], Dana Tomescu[2,3], Mihai Popescu[2,3], Ecaterina Scărlătescu[2,3], Gabriela Droc[2,4], Lavinia Brezeanu[2,4], Ruxandra Tulbure[5], Dan Corneci[6,7], Narcis Valentin Tanase[6], Dan Mihai Lantis[6], Augustin Tudose[6], Cristian Plesa[6], Mădălina Duțu[2,7], Silvius Negoiță[2,7], Emanuel Moisa[2,7], Cosmin Balan[8], Cornel Robu[8], Teodora Bute[8], Daniela Filipescu[2,9], Mihai-Gabriel Stefan[9], Bogdan Prodan[9], Cornelia Predoi[9], Raluca Goicea[9], Camelia Călin[10], Raluca Havriliuc[11], Lelia Iliescu[12], Ioana Simion[13], Delia Stanciu[14], Cătălin Macovei[15], Florin-Nicolae Tainǎ[15], Viorica-Daniela Mitescu[15], Tudor Vladoiu[15], Andrei Cuibari[15,] Andra Hagiu[15], Manuela Dan[15], Adrian Stănculea[16], Cristina Petrișor[17], Daniela Ionescu[18], Bianca Burghelea[19], Daniela Prunoiu[20], Aurelia Medan[21], Mihai Soare[22], Carolina Susanu[23], Simona Gălbineanu[24], Anamaria Hărăbor[25], Corina Manole Palivan[26], Lucian Horhotă[27], Iasmina Salim[28], Liviu Antohi[29], Ionuț Soare[30], Claudia Iuga[31], Mircea Stoian[32], Gabriela Nacu[33], Viorel Vereș[34], Gordana Boanță[35], Elena Chircov[36], Georgeta Gugonea[37], Mihaiela Marian[38], Alice Dragoescu[39], Janos Szederjesi[40], Sanda Copotoiu[40], Ioana Grigoras[41,42], Irina Ristescu[41,42], Constantin Bodolea[43], Horatiu Moldovan[44,45], Alexandru-Florin Rogobete[46,47], Dorel Sandesc[46,47], Raed Arafat[48].

[1] Anaesthesiology and Intensive Care Department, Emergency Hospital, 8 Floreasca Road, 014461, Bucharest, Romania
[2] Anesthesiology and Intensive Care Department, Carol Davila University of Medicine and Pharmacy, 8 Eroii Sanitari Blvd., 050474, Bucharest, Romania
[3] Anaesthesiology and Intensive Care Department III, Fundeni Clinical Institute, 258 Fundeni Road, 022328, Bucharest, Romania
[4] Anaesthesiology and Intensive Care Department I, Fundeni Clinical Institute, 258 Fundeni Road, 022328, Bucharest, Romania
[5] Anaesthesiology and Intensive Care Department II, Fundeni Clinical Institute, 258 Fundeni Road, 022328, Bucharest, Romania
[6] Anaesthesiology and Intensive Care Department I, Central Military University Emergency Hospital, 134 Plevnei Road, 010825, Bucharest, Romania
[7] Anaesthesiology and Intensive Care Department, Elias Emergency University Hospital, 17 Mărăști Blvd. 11461, Bucharest, Romania
[8] Cardiac Anesthesiology and Intensive Care Department I, Emergency Institute for Cardiovascular Diseases Prof. Dr. C. C. Iliescu, 258 Fundeni Road, 022328, Bucharest, Romania
[9] Cardiac Anesthesiology and Intensive Care Department II, Emergency Institute for Cardiovascular Diseases Prof. Dr. C. C. Iliescu, 258 Fundeni Road, 022328, Bucharest, Romania
[10] Anaesthesiology and Intensive Care Department, Sf Ioan Emergency Hospital, 13 Vitan-Bârzești Road, 042122, Bucharest, Romania
[11] Anaesthesiology and Intensive Care Department, Clinical Hospital Nicolae Malaxa, 12 Vergului Road, 02441, Bucharest, Romania
[12] Anaesthesiology and Intensive Care Department I, Clinical Hospital Colentina, 19-21 Ștefan cel Mare Road, 020125, Bucharest, Romania
[13] Anaesthesiology and Intensive Care Department II, Clinical Hospital Colentina, 19-21 Ștefan cel Mare Road, 020125, Bucharest, Romania
[14] Anaesthesiology and Intensive Care Department, Clinical Hospital of Infectious and Tropical Diseases Victor Babeș, 281 Mihai Bravu Road, 030303, Bucharest, Romania
[15] Anaesthesiology and Intensive Care Department, National Institute for Infectious Diseases Matei-Balș, 1 Dr. Calistrat Grozovici Street, 021105, Bucharest, Romania
[16] Anaesthesiology and Intensive Care Department, Clinical Emergency Hospital of Plastic Surgery, Repairs and Burns, 218 Griviței Road, 010713, Bucharest, Romania

[17] Anaesthesiology and Intensive Care Department, Cluj-Napoca County Clinical Emergency Hospital, 3-5 Clinicilor Street, 400000, Cluj-Napoca, Romania
[18] Anaesthesiology and Intensive Care Department, Regional Institute of Gastroenterology and Hepatology Prof. Dr. Octavian Fodor, 19 Coitorilor Street, Cluj-Napoca, Romania
[19] Anaesthesiology and Intensive Care Department, Clinical Rehabilitation Hospital Cluj-Napoca, 46-50 Viilor Street, 400066, Cluj-Napoca, Romania
[20] Anaesthesiology and Intensive Care Department, Câmpulung Municipal Hospital, 8 Doctor Costea Street, 115100, Câmpulung, Romania
[21] Anaesthesiology and Intensive Care Department, Bistrița Emergency County Hospital, 43 General Grigore Bălan Blvd, 420094, Bistrița, Romania
[22] Anaesthesiology and Intensive Care Department I, Brăila Emergency County Hospital, 2 Buzăului Road, 810325, Brăila, Romania
[23] Anaesthesiology and Intensive Care Department II, Brăila Emergency County Hospital, 2 Buzăului Road, 810325, Brăila, Romania
[24] Anaesthesiology and Intensive Care Department, Brașov Clinical Emergency County Hospital, 25 București Road, 500326, Brașov, Romania
[25] Anaesthesiology and Intensive Care Department, Clinical Hospital of Obstetrics and Gynaecology Buna Vestire, 99 Nicolae Alexandrescu Street, 800151, Galați, Romania
[26] Anaesthesiology and Intensive Care Department,Clinical Emergency Hospital St. Apostol Andrei, 177 Brăilei Street, 800578, Galați, Romania
[27] Anaesthesiology and Intensive Care Department, Bolintin Vale City Hospital, 5 Republicii Street, 085100, Bolintin-vale, Romania
[28] Anaesthesiology and Intensive Care Department, Lupeni Municipal Hospital, Tudor Vladimirescu Street, 335600, Lupeni, Romania
[29] Anaesthesiology and Intensive Care Department, Clinical Hospital Dr. C. I. Parhon, 50 Carol I Blvd, 700503, Iași, Romania
[30] Anaesthesiology and Intensive Care Department, Ilfov Emergency Clinical County Hospital, 49-51 Basarabia Blvd. 22104, Bucharest, Romania
[31] Anaesthesiology and Intensive Care Department, Orșova Municipal Hospital, 34 Porțile de Fier Street, 225200, Orșova, Romania
[32] Anaesthesiology and Intensive Care Departament, Târgu Mureș Clinical County Hospital, 1 Gheorghe Marinescu Street, 540136, Târgu Mureș, Romania
[33] Anaesthesiology and Intensive Care Departament, Ploiești Emergency County Hospital, 100 Găgeni Street, 100137, Ploiești, Romania
[34] Anaesthesiology and Intensive Care Departament, Zalău Emergency County Hospital, 67 Simion Bărnuțiu Street, 917151, Zalău, Romania
[35] Anaesthesiology and Intensive Care Departament, Timișoara Emergency Municipal Hospital, 5 Gheorghe Dima Street, 300254, Timișoara, Romania
[36] Anaesthesiology and Intensive Care Departament, Tulcea Emergency County Hospital, 32 1848 Street, 820180, Tulcea, Romania
[37] Anaesthesiology and Intensive Care Departament, Clinical Hospital of Nephrology Dr. Carol Davila, 4 Griviței Road, 010731, Bucharest, Romania
[38] Anaesthesiology and Intensive Care Department, Tarnaveni Municipal Hospital, 2 Victor Babes Street, 545600, Tarnaveni, Romania
[39] Anaesthesiology and Intensive Care Department, Craiova Emergency County Hospital, 1 Tabaci Street, 200642, Craiova, Romania
[40] Anaesthesiology and Intensive Care Department, George Emil Palade University of Medicine, Pharmacy, Science, and Technology, 38 Gh. Marinescu Street, 540142, Târgu Mureș, Romania
[41] Anaesthesiology and Intensive Care Department, Grigore T. Popa University of Medicine and Pharmacy, 16 Universității Street, 700115 Iași, Romania

[42] Anaesthesiology and Intensive Care Department, Regional Institute of Oncology, 2-4 Henri Berthelot Street, 700483, Iași, Romania
[43] Anaesthesiology and Intensive Care Department II, Iuliu Hațieganu University of Medicine and Pharmacy, 8 Victor Babeș Street, 400012, Cluj-Napoca, Romania
[44] Cardiovascular Surgery Department, Emergency Hospital, 8 Floreasca Road, 014461, Bucharest, Romania
[45] Cardiovascular Surgery Department, Carol Davila University of Medicine and Pharmacy, 8 Eroii Sanitari Blvd., 050474, Bucharest, Romania
[46] Anaesthesiology and Intensive Care Department, Emergency County Hospital Pius Brinzeu, 10 Iosif Bulbuca Blvd., Timișoara, Romania
[47] Anaesthesiology and Intensive Care Department, Victor Babeș University of Medicine and Pharmacy, 2 Eftimie Murgu Sq, 300041, Timișoara, Romania
[48] Department for Emergency Situations, Ministry of Internal Affairs, 1 Revolution Sq, 030167, Bucharest, Romania
